# Primary primitive neuroectodermal tumor: An unusual cause of right ventricular intracavitary obstruction in a child

**DOI:** 10.4103/0974-2069.43884

**Published:** 2008

**Authors:** Ajit Thachil, Anita Saxena, Ujjwal K. Choudhary, Ruma Ray

**Affiliations:** 1 Department of Cardiology, All India Institute of Medical Sciences, New Delhi, India; 2 Department of Cardiothoracic and Vascular Surgery, All India Institute of Medical Sciences, New Delhi, India; 3 Department of Pathology, All India Institute of Medical Sciences, New Delhi, India

**Keywords:** Cardiac masses, neuroectodermal tumor, cardiac failure

## Abstract

A six-year-old boy presented with a brief history suggestive of right heart failure. Investigations revealed a mass filling almost the entire right ventricle. Palliative resection of the mass was done. The operative specimen revealed a primary primitive neuroectodermal tumor of the heart, the first of its kind reported in the pediatric age group.

## CASE REPORT

A six-year-old boy presented with a one month history of abdominal distension and facial puffiness. There was a brief episode of low-grade fever with constitutional symptoms two weeks prior to the current illness. Physical examination revealed jugular venous distention, prominent hepatomegaly, and ascites. His ECG was normal, while the chest X-ray showed cardiomegaly with right atrial enlargement and normal pulmonary vascularity.

**Figure 1:**
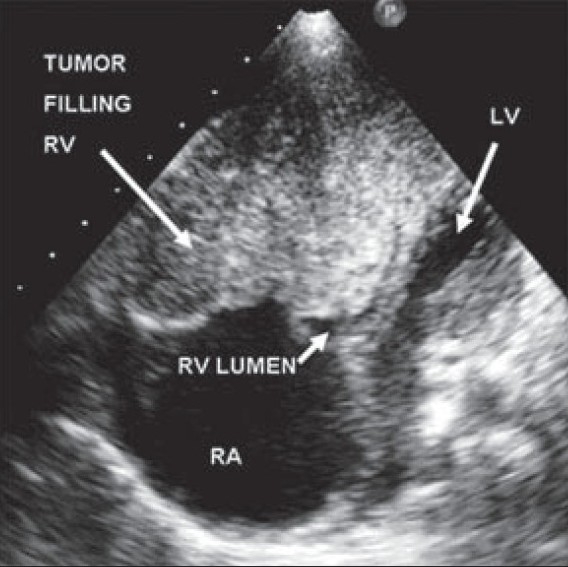
Echocardiogram in apical 4 – chamber view; RA, right atrium; RV, right ventricle; LV, left ventricle

**Figure 2:**
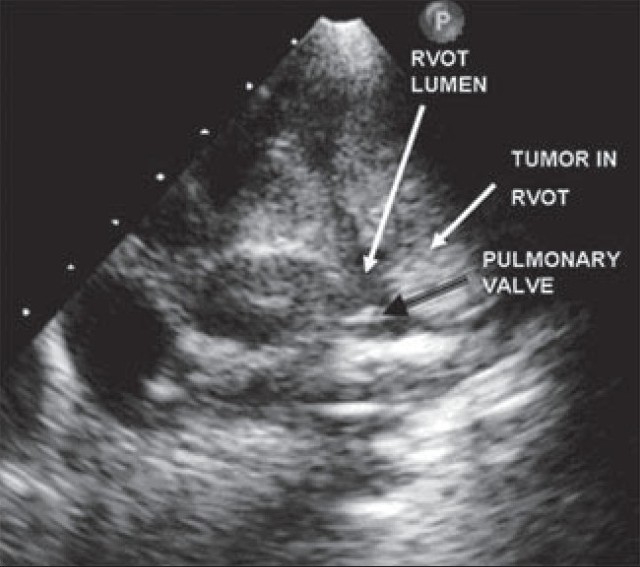
Extension of the tumor into the RVOT showing the tumor filling the right ventricle in parasternal short axis view; RVOT, right ventricular outflow tract

Transthoracic echocardiography revealed a large solid mass of inhomogeneous echo texture filling the entire right ventricle including the outflow tract, leaving a residual lumen of only a few millimeters [Figures 1 and 2]. This mass was indistinguishable from the myocardium, and delimited by the tricuspid and pulmonary valves. The right atrium was enlarged and left-sided cardiac chambers were compressed. There was a small pericardial effusion. Systemic evaluation did not show any evidence of extracardiac disease. A provisional diagnosis of rhabdomyosarcoma was considered. Cardiac MRI did not add to this diagnosis. The mass was not biopsied as an urgent palliative resection was planned in view of the obstructive symptoms. Considering the extent of the tumor, it was approached through an atriotomy as well as through the pulmonary artery. The tumor was entirely intracavitary, its extent being as suggested by the echocardiographic appearance. The entire mass was resected and a Glenn shunt was performed. The foramen ovale was left patent. The immediate postoperative period was uneventful. However, on the first postoperative day, the patient expired, likely due to an air embolism.

**Figure 3:**
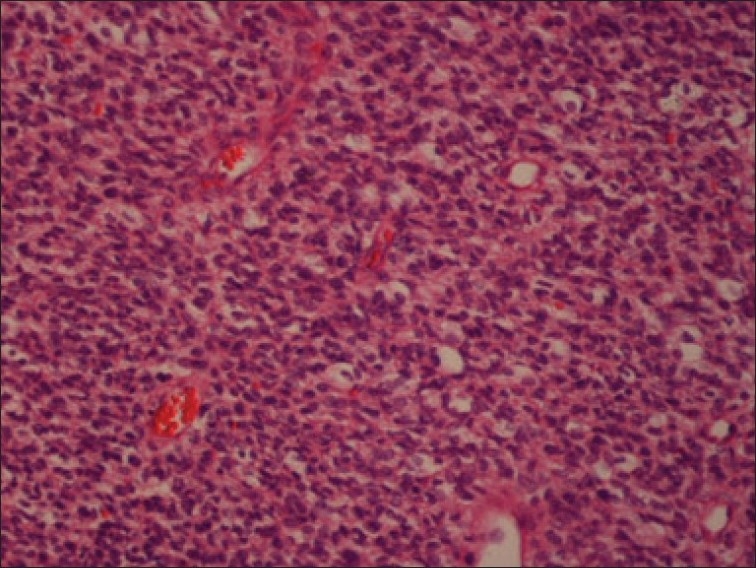
Histology – H and E stain showing a small round cell tumor

**Figure 4:**
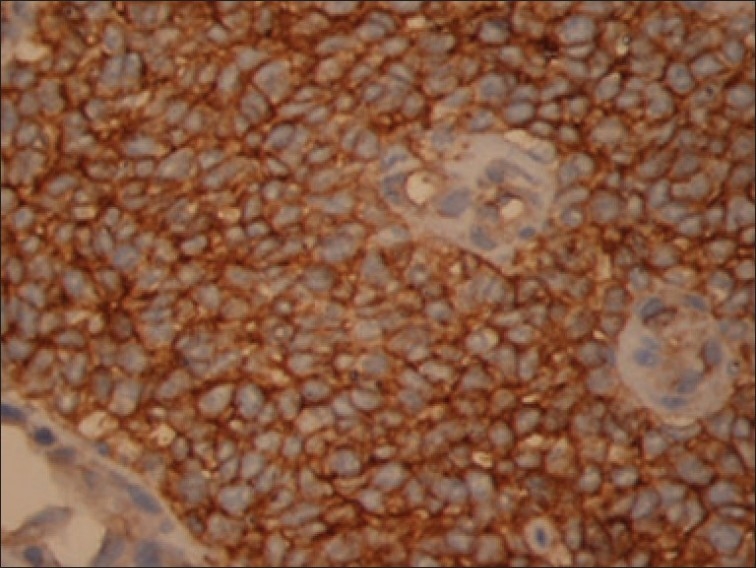
Immunocytochemistry – tumor cells show membrane positivity for MIC-2 (CD99), suggesting a primitive neuroectodermal tumor

Histology and histochemistry of the operative specimen revealed a malignant small round cell tumor [Figure 3] with abundant intracytoplasmic glycogen. Immunocytochemistry was positive for vimentin and MIC2 (CD99), suggesting a primitive neuroectodermal tumor (PNET) [Figure 4]. Negativity for smooth muscle actin, desmin, and myogenin ruled out the commoner possibility of a rhabdomyosarcoma. The other two malignant small round cell cardiac tumors ( please delete the intervening commas) cell cardiac tumors viz. lymphoblastic lymphoma and neuroblastoma were ruled out by virtue of negativity for leucocyte common antigen, chromogranin and synaptophysin. Thus, a final diagnosis of a PNET of the myocardium was made.

## DISCUSSION

Cardiac tumors usually present with features related to the site of involvement rather than the specific tumor *per se*. As with other age groups, secondary cardiac tumors, such as Non- Hodgkin's lymphoma, leukemia, and neuroblastma are commoner than primary tumors in children. Malignant myocardial tumors represent less than 10% of primary cardiac tumors in pediatric patients.[1,2] The commonest malignant tumors in this age group are sarcomas, usually angiosarcoma and rhabdomyosarcoma.

Cardiac involvement by primary PNETs, commonly a tumor of the extremities, was first reported in 1996.[3] Four more reports followed, all cases so far having been reported in adults.[4–7] This is the first ever report of a cardiac PNET in the pediatric age group. Cardiac PNETs are aggressive tumors with rapid growth. An early cardiac transplantation, if feasible, may be the best treatment option. If not, palliative resection may have to be considered, as was done in our case
